# Supplementary material for: GC-MS Profiling of Ethanol-Extracted Polyherbal Compounds from Medicinal Plant (Citrullus colocynthis, Curcuma longa, and Myristica fragrans): In Silico and Analytical Insights into Diabetic Neuropathy Therapy via Targeting the Aldose Reductase
Source: Curr Issues Mol Biol. 2025 Jan 23;47(2):75. doi: 10.3390/cimb47020075 (PMC11854889; doi:10.3390/cimb47020075)
Supplement: Supplementary file 1 [file cimb-47-00075-s001.zip › cimb-3389720-supplementary.pdf]

GC-MS Profiling, MD simulation, and Pharmacokinetic evaluation of the Polyherbal Extract with Potential Against Diabetic Neuropathy.

Supplementary Figure S1: Mass spectra of identified bioactive compound from PHE with their chemical structure

A

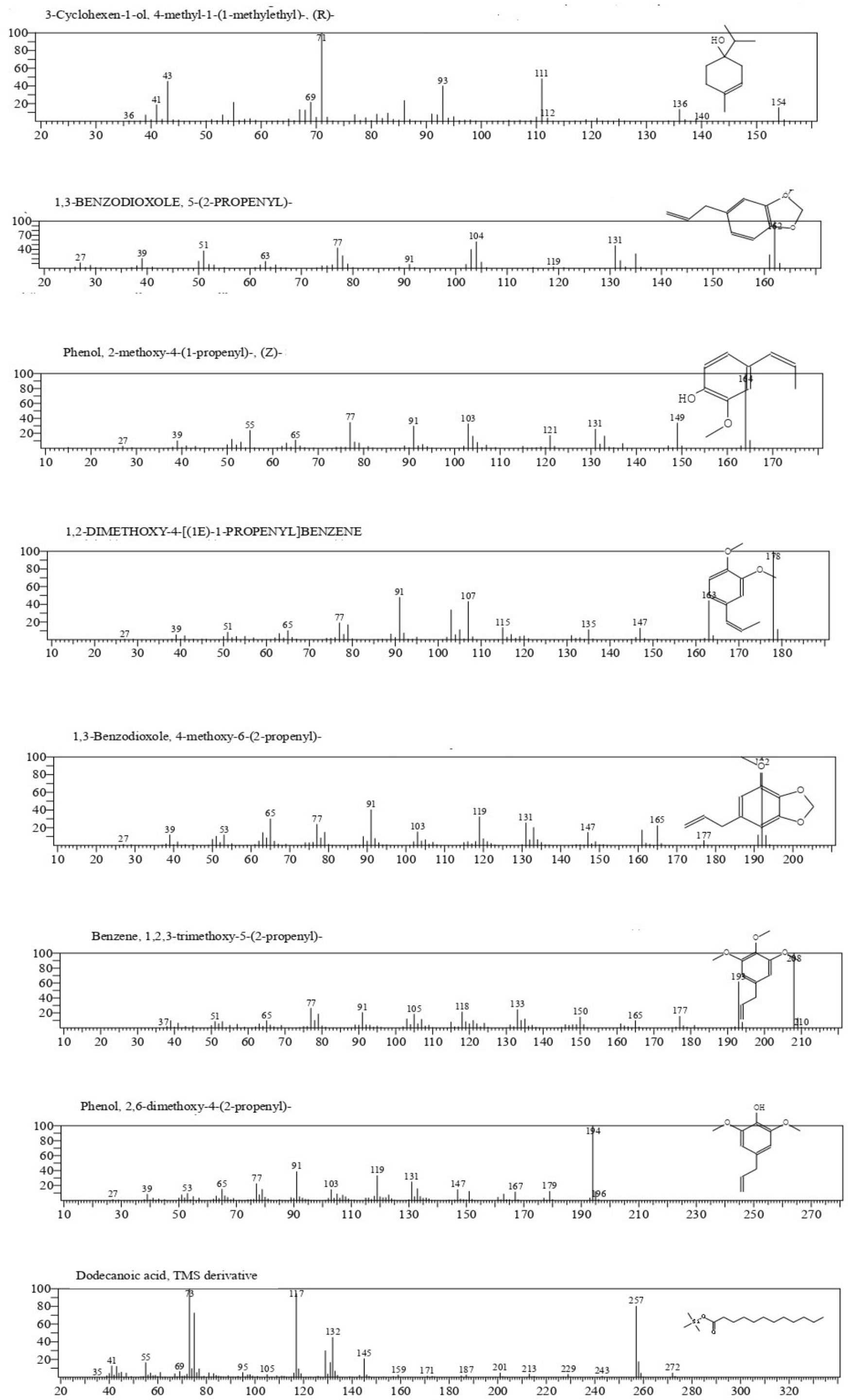

Figure S1. A: Mass spectra of identified bioactive compound group 1 from PHE with their chemical structure

B

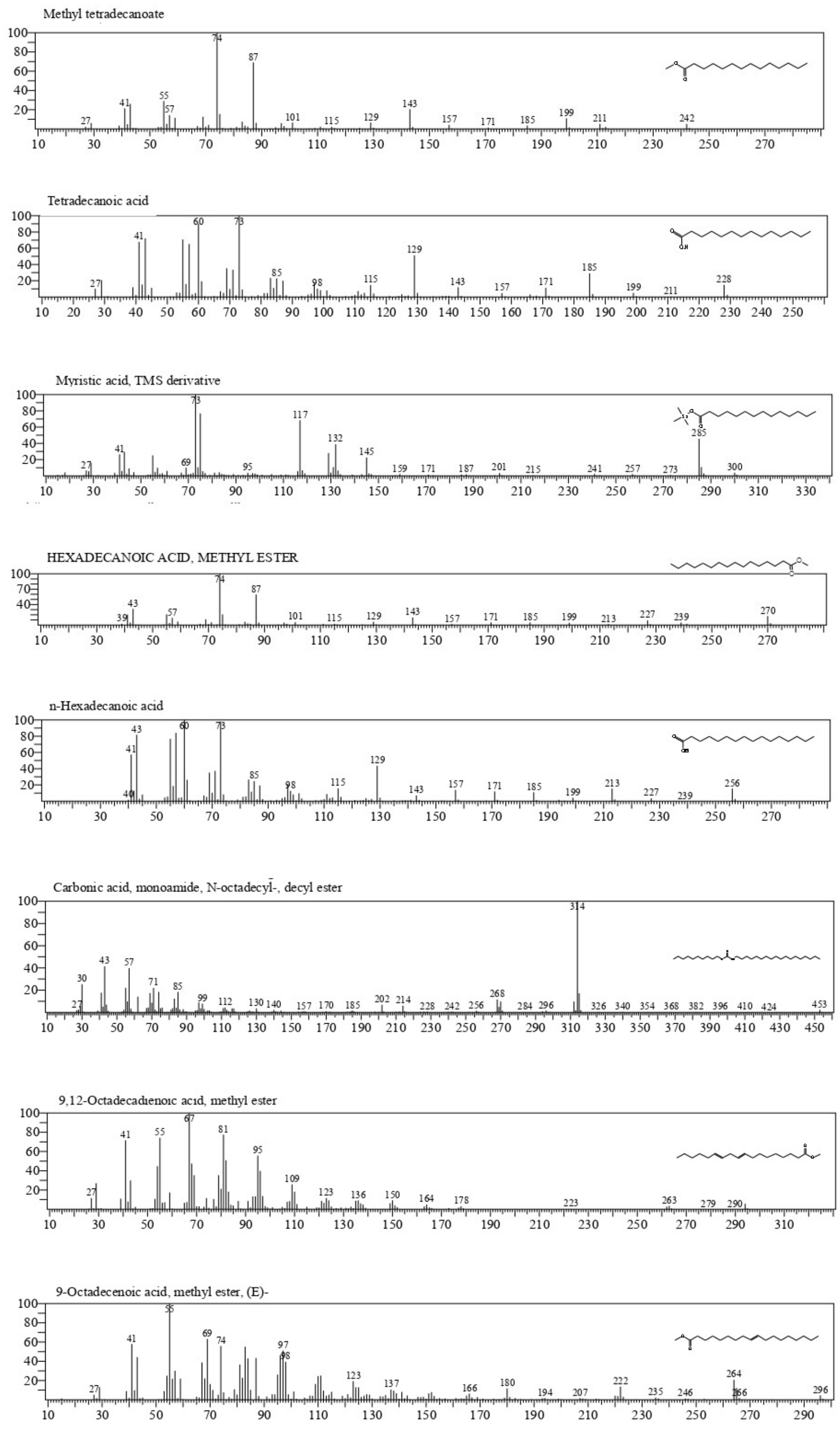

Figure S1. B: Mass spectra of identified bioactive compound group 2 from PHE with their chemical structure

C

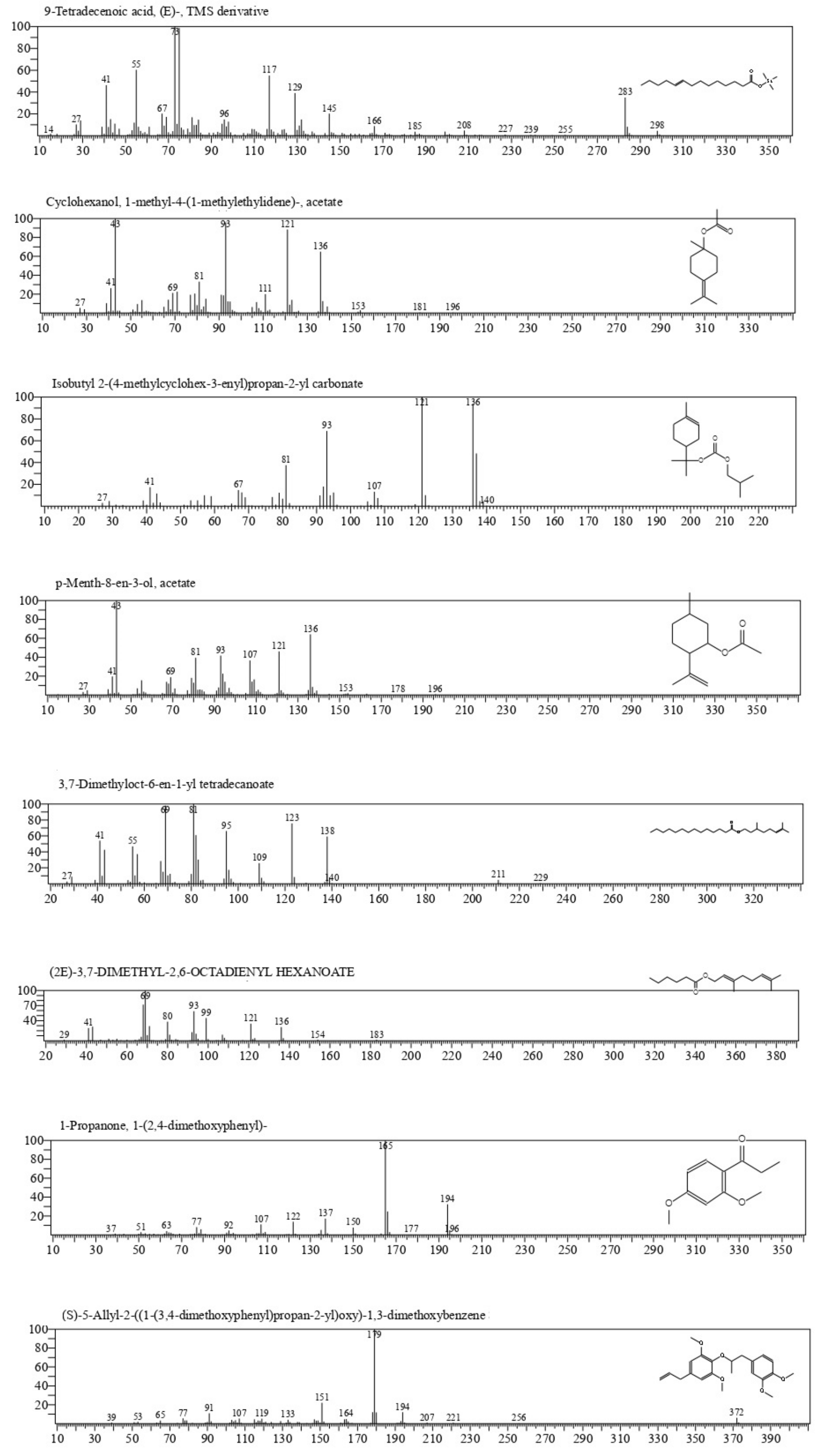

Figure S1. C: Mass spectra of identified bioactive compound group 3 from PHE with their

chemical structure

D

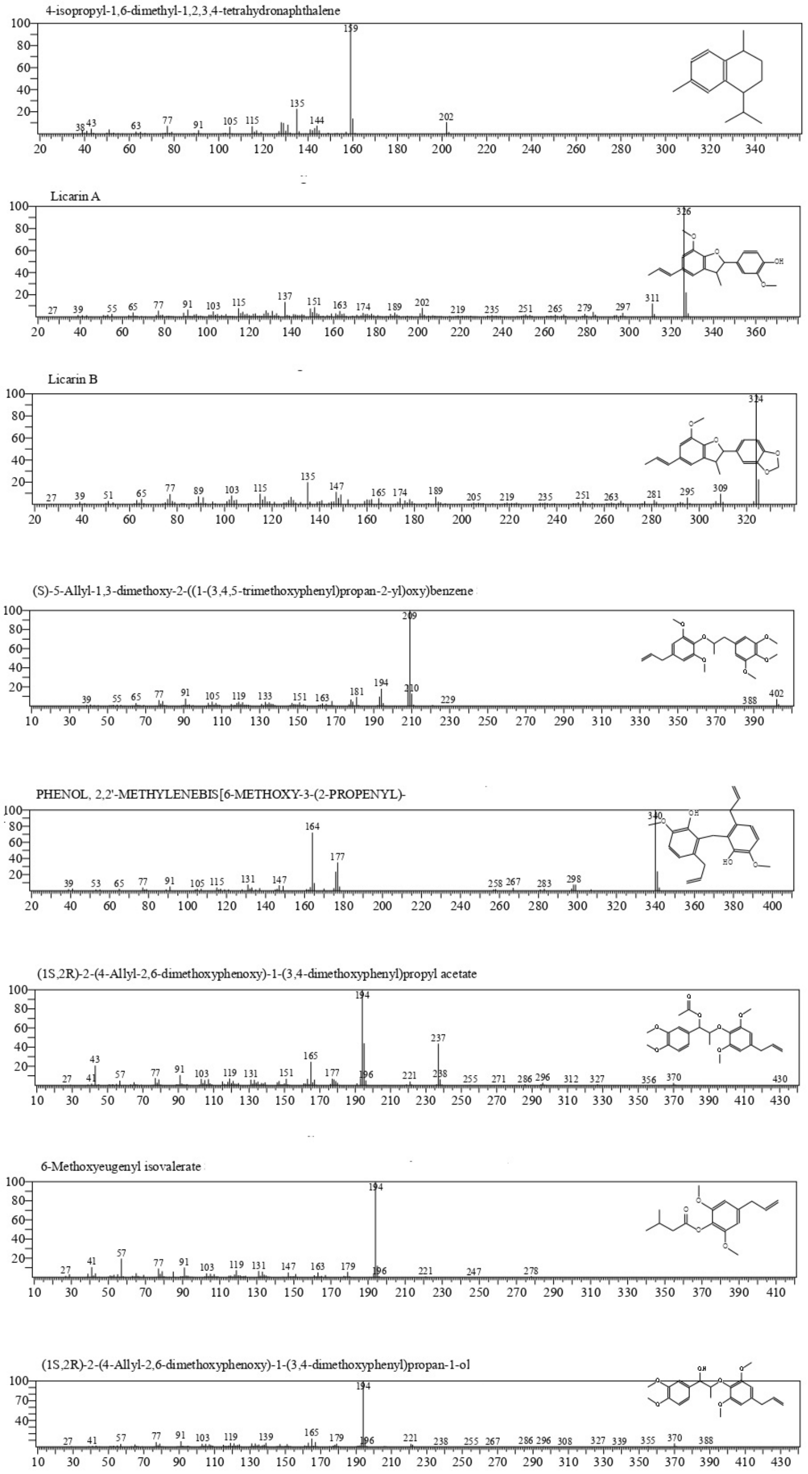

Figure S1. D: Mass spectra of identified bioactive compound group 4 from PHE with their chemical structure

E

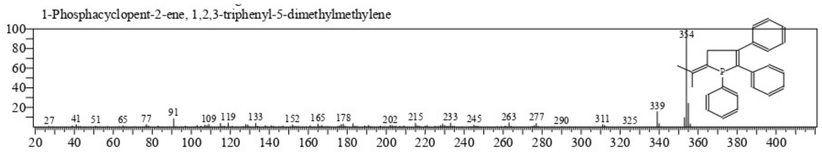

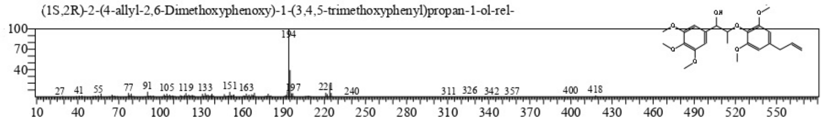

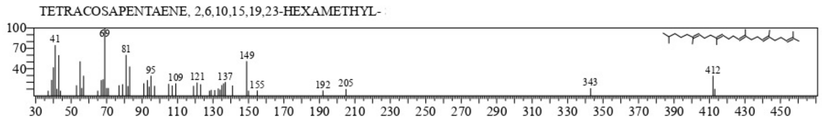

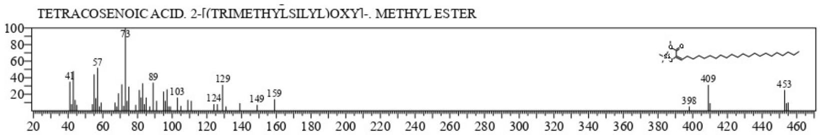

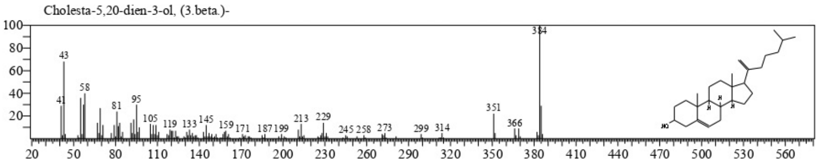

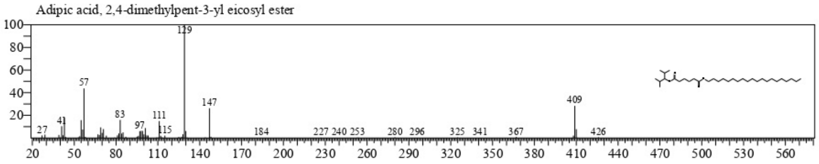

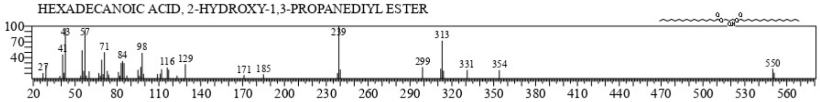

Figure S1. E: Mass spectra of identified bioactive compound group 5 from PHE with their chemical structure

Supplementary Table S1. Screening of phytochemical compounds according to Lipinski Rule.
Lipinski (0) means does not violate the Lipinski rule and Lipinski (1) violate the Lipinski factors. H (hydrogen bond), log P indicate lipophilicity, M.W (Molecular Weight).

S.No	Compound name	PubChem ID	M.W	LogP	H bond donor	Hbond acceptor	Lipinski
1.	Tetradecanoic acid	11005	228.37	6.48	1	2	1
2.	TETRACOSENOIC ACID, 2-[(TRIMETHYLSILYL)OXY	5366389	468.83	7.48	0	3	1
3.	TETRACOSAPENTAENE, 2,6,10,15,19,23-HEXAMETHYL	5366021	412.73	2.45	0	0	0
4.	p-Menth-8-en-3-ol, acetate	94579	196.29	2.65	0	2	0
5.	Phenol, 2-methoxy-4-(1-propenyl)-, (Z)-	1549041	164.2	6.23	1	2	1
6.	Phenol, 2,6-dimethoxy-4-(2-propenyl)-	226486	194.23	5.98	1	3	1
7.	PHENOL, 2,2'-METHYLENEBIS[6-METHOXY-3-(2-PR	630963	340.41	6.08	2	4	1
8.	HEXADECANOIC ACID, 2-HYDROXY-1,3-PROPANEDIYL ESTER	985	256.42	7.78	1	2	1
9.	Myristic acid, TMS derivative	519592	300.55	5.78	0	2	1
10.	Methyl tetradecanoate	31284	242.4	6.98	0	2	1
11.	Licarin B	6441061	324.37	6.22	0	4	1
12.	Licarin A	5281836	326.39	6.45	1	4	1
13.	Isobutyl 2-(4-methylcyclohex-3-enyl)propan-2-yl carbonate	91711074	254.37	3.67	0	3	0
14.	HEXADECANOIC ACID, METHYL ESTER	8181	270.45	7.45	0	2	1
15.	Dodecanoic acid, TMS derivative	521640	272.5	5.67	0	2	1

16.	Cyclohexanol, 1-methyl-4-(1-methylethylidene)-, acetate	82480	196.29	5.98	1	2	1
17.	Carbonic acid, monoamide, N-octadecyl-, decyl ester	85800427	453.78	6.89	0	2	1
18.	Benzene, 1,2,3-trimethoxy-5-(2-propenyl)-	10248	208.25	2.89	0	3	0
19.	Adipic acid, 2,4-dimethylpent-3-yl eicosyl ester	91713311	524.86	7.63	0	4	2
20.	9-Tetradecenoic acid, (E)-, TMS derivative	5366416	298.54	5.67	0	2	1
21.	9-Octadecenoic acid, methyl ester, (E)-	5280590	296.49	5.98	0	2	1
22.	9,12-Octadecadienoic acid, methyl ester	5284421	294.47	6.34	0	2	1
23.	6-Methoxyeugenyl isovalerate	23727651	278.34	6.32	0	4	1
24.	4-isopropyl-1,6-dimethyl-1,2,3,4-tetrahydronaphthalene	10224	202.34	3.16	1	0	0
25.	Cyclohexanol, 1-methyl-4-(1-methylethylidene)-, acetate	5325830	154.25	5.78	0	1	1
26.	3,7-Dimethyloct-6-en-1-yl tetradecanoate	85793156	366.62	2.32	0	2	0
27.	1-Propanone, 1-(2,4-dimethoxyphenyl)-	70020	194.23	2.31	0	3	0
28.	1,3-BENZODIOXOLE, 5-(2-PROPENYL)-	5144	162.19	5.44	0	2	1
29.	1,3-BENZODIOXOLE, 5-(2-PROPENYL)-	4276	192.21	5.89	0	3	1
30.	(S)-5-Allyl-2-((1-(3,4-dimethoxyphenyl)propan-2-yl)oxy)-1	91727291	372.45	4.25	0	5	0
31.	(S)-5-Allyl-1,3-dimethoxy-2-((1-(3,4,5-trimethoxyphenyl)p	91734155	402.48	5.67	0	6	1

32.	(2E)-3,7-DIMETHYL-2,6-OCTADIENYL HEXANOATE	5365992	252.39	5.33	1	2	1
33.	(1S,2R)-2-(4-Allyl-2,6-dimethoxyphenoxy)-1-(3,4-dimetho	71437670	418.48	5.90	0	7	1
34.	(1S,2R)-2-(4-allyl-2,6-Dimethoxyphenoxy)-1-(3,4,5-trimethoxyphenyl) propan-1-ol-rel-	10477119	354.42	4.75	1	0	0
35.	3-Cyclohexen-1-ol, 4-methyl-1-(1-methylethyl)-, (R)-	5325830	154.25	5.2	2	5	0
36.	Cholesta-5,20-dien-3-ol, (3.beta.)-	15429531	384.6	5.88	1	3	1
37.	HEXADECANOIC ACID, 2-HYDROXY-1,3-PROPANEDIYL ESTER	68149	568.9	5.43	0	2	1
38.	1-Phosphacyclopent-2-ene, 1,2,3-triphenyl-5-dimethylmeth	631947	354.4	5.78	2	2	1
39.	(1S,2R)-2-(4-Allyl-2,6-dimethoxyphenoxy)-1-(3,4-dimethoxyphenyl)propyl acetate	9932416	430.5	6.09		3	1
